# The Asparaginyl Endopeptidase Legumain: An Emerging Therapeutic Target and Potential Biomarker for Alzheimer’s Disease

**DOI:** 10.3390/ijms231810223

**Published:** 2022-09-06

**Authors:** Mingke Song

**Affiliations:** Department of Pharmacology and Chemical Biology, Institute of Medical Sciences, Shanghai Jiao Tong University School of Medicine, 280 South Chongqing Road, Shanghai 200025, China; mksong@sjtu.edu.cn; Tel.: +86-21-6384-6590 (ext. 776794); Fax: +86-21-6467-4721

**Keywords:** asparaginyl endopeptidase, legumain, Alzheimer’s disease, amyloid-β protein, tau, therapeutic targets, biomarker, live brain imaging, neurological diseases

## Abstract

Alzheimer’s disease (AD) is incurable dementia closely associated with aging. Most cases of AD are sporadic, and very few are inherited; the pathogenesis of sporadic AD is complex and remains to be elucidated. The asparaginyl endopeptidase (AEP) or legumain is the only recognized cysteine protease that specifically hydrolyzes peptide bonds after asparagine residues in mammals. The expression level of AEPs in healthy brains is far lower than that of peripheral organs. Recently, growing evidence has indicated that aging may upregulate and overactivate brain AEPs. The overactivation of AEPs drives the onset of AD through cleaving tau and amyloid precursor proteins (APP), and SET, an inhibitor of protein phosphatase 2A (PP2A). The AEP-mediated cleavage of these peptides enhances amyloidosis, promotes tau hyperphosphorylation, and ultimately induces neurodegeneration and cognitive impairment. Upregulated AEPs and related deleterious reactions constitute upstream events of amyloid/tau toxicity in the brain, and represent early pathological changes in AD. Thus, upregulated AEPs are an emerging drug target for disease modification and a potential biomarker for predicting preclinical AD. However, the presence of the blood–brain barrier greatly hinders establishing body-fluid-based methods to measure brain AEPs. Research on AEP-activity-based imaging probes and our recent work suggest that the live brain imaging of AEPs could be used to evaluate its predictive efficacy as an AD biomarker. To advance translational research in this area, AEP imaging probes applicable to human brain and AEP inhibitors with good druggability are urgently needed.

## 1. Introduction

Asparaginyl endopeptidases (AEPs; EC 3.4.22.34) are the C13 family of cysteine proteases that specifically cleave peptide bonds at their carboxyl-terminal side of asparagine (Asn) or asparate (Asp) residues. AEPs were first discovered in *Schistosoma mansoni* and leguminous seeds over 30 years ago [1,2], and are also known as legumains or vacuolar processing enzymes that are important for seed storage and plant defense responses [2,3]. Later identified in human, murine, and other mammalian organs, AEPs are activated in the acidic environment of lysosomes, and primarily perform proteolytic enzyme activity to hydrolyze peptide bonds [4].

Various AEP isoforms exist in plants, but only one functional isoform was identified in mammals, including humans, and none in bacteria [5,6]. In plants, some AEP isoforms function as transpeptidases (ligases) that catalyze peptide cyclization to help in forming new peptide bonds [5,6]. In murine T cells and dendritic cells, AEPs are required for the cleavage of cathepsin L, toll-like receptor 7 (TLR7), and TLR9 to perform signaling transduction [7,8,9,10]. In a human B cell line, AEPs participated in Class II MHC antigen (Ag) presentation through processing a microbial Ag [11]. However, a different report argued that AEPs were not essential for Class II MHC maturation in murine Ag-presenting cells (APCs), including primary B cells and bone-marrow-derived dendritic cells [12]. The functional properties of AEPs in normal physiology remain largely unknown [13,14], while increasing attention has been drawn towards the pathogenic roles of AEPs in neurological diseases. This review presents the latest advances in AEP-related neurodegeneration studies and their potential as a modification target for Alzheimer’s disease (AD).

## 2. Distribution and Activation of Mammalian AEPs

In human and murine bodies, AEPs display broad tissue distribution, highly expressed in the kidneys, placenta, spleen, bladder, lungs, and thyroid and lymph nodes, whereas relatively less in the bone marrow, pancreas, skin, heart, and the nervous system [13] (National Center for Biotechnology Information: https://www.ncbi.nlm.nih.gov/gene/5641 (accessed on 12 August 2022)). Our recent data obtained from samples of mice show that the enzymatic activity of AEPs in the brain was almost 20 times lower than that in the kidneys or liver [15]. Accumulating data indicate that many tumors and nervous system diseases overexpress AEPs [16,17]. Having 433 amino acids, human AEPs are encoded by the LGMN gene located on chromosome 14q32.12 [18]. Murine LGMN gene is on chromosome 12, 51.45 and encodes 435 amino acids that share 83% homology with that of the human protein [19]. AEPs are initially assembled as proenzymes in endoplasmic reticulum (ER) bodies, translocated via Golgi to the endosome when necessary, and activated to the mature form in the acidic environment of the lysosome. Both AEP precursors and matured AEPs are found in the cytoplasm and nucleus, on the cell surface and outside of a cell, particularly under certain pathological conditions, e.g., ischemia, neurodegeneration, and tumorigenesis [13].

AEP precursors contain a caspaselike catalytic domain and a C-terminal death domainlike fold (legumain stabilization and activity modulation, LSAM domain), linked by an activation peptide (AP). There are three amino acid residues, His148, Cys189, and Asn42, in the catalytic domain, which is critical for AEPs to perform a hydrolytic function. AEP precursors are enzymatically inactive at neutral pH because substrate access is impeded by the LSAM on substrate binding sites [20]. At an acidic pH, autocatalytic processing occurs in AEP precursors (53 kDa), which is self-cleavage at Asn323 (pH ≤ 5.5) of the C-terminal and at Asp25 or Asp21 (pH ≤ 4.5) of the N-terminal, generating 46 and 47 kDa intermediate AEP species, respectively. The cleavage at the Asn323 of the C-terminal is essential for AEP activation [21], followed by the additional trimming of the 46 kDa intermediate AEP via unrevealed proteases to generate the mature form of AEPs (36 kDa). This autocatalytic cleavage processing releases the LSAM domain and AP, leading the substrate binding sites to be accessible. For more details on this pH-induced (electrostatic) autoproteolytic processing and AEP activation mechanism, please refer to other excellent reports and reviews [13,20,22,23,24].

Acidic pH condition is essential for matured AEPs to maintain protease activity; uncomplexed AEPs are irreversibly inactivated by neutral pH. However, it is likely that the activity of AEPs can be stabilized in the neutral pH milieus of cytosol and cell membrane if matured AEPs are bound by integrins or TRAF6 and HSP90α [13]. AEPs can be inhibited to varying extents by human type 2 cystatins C, E/M, and F [25,26], which had been identified as endogenous inhibitors of cathepsins, members of the family of papainlike cysteine proteases.

## 3. Proteolytic Substrates of Mammalian AEPs

Characterized by a conserved His–Gly–spacer–Ala–Cys catalytic motif, AEPs belong to the family of clan CD enzymes with other members, including caspases, clostripains, gingipains, and separases [27]. Intriguingly, AEPs can be inhibited by cystatins, and share similar protein degradation ability with cysteine cathepsins, and similar endolysosomal localization and pH-dependent maturation pattern [28]. Therefore, AEPs are biochemically related to cathepsins even though they have no sequence homology. These features bring extra difficulty to exploring potent and selective AEP substrates and inhibitors, which need to be distinguished between AEPs and cathepsins or caspases.

When acting as proteolytic enzymes, mammalian AEPs selectively cleave peptide bonds bearing Asn or Asp residues at the so-called P1 position of substrates [21,29]. Z–Ala–Ala–Asn–AMC, the first synthetic substrate of AEPs, appeared about 30 years ago, and its synthesis was based on the P3–P2–P1 sequences Ala–Ala–Asn [30]. It is used as the gold standard for fluorometric assays of AEP activity; the specificity of AEPs for this substrate enables researchers to detect AEP activity in extracts of various tissues and screen AEP inhibitors [27]. More substrates with the Ala–Ala–Asn scaffold were continuously designed for the colorimetric assay or region-specific histochemical staining of AEPs. Substrate-based tools, prodrugs, and probes incessantly emerged for the purpose of investigating tumor diagnosis, antitumor therapy, and AEP-activity-based live imaging [27,31].

Besides exogenous substrates, numerous endogenous substrates of AEPs have been identified in diverse mammalian tissues. In the kidney-proximal tubule cells of mice, AEPs process lysosomal proteases cathepsins B and H through cleaving peptide bonds containing Asn residues at the P1 position, and cleave cathepsin L at the site of Asp residues [32]. The AEP-mediated cleavage of cathepsin L also occurs in splenic, thymic, and bone-marrow-derived dendritic cells as well as human CD4^+^ T cells [12,33]. In these cells, AEP is required for cathepsins B, H, and L to be converted from proenzymes into mature two-chain forms. In the absence of AEPs, cathepsins B, H, and L are compensatorily induced to escalate activities or tend to use the mature single-chain forms to carry out essential functions. Cathepsin inhibitors cystatin C and E/M, in addition to functioning as AEP inhibitors, are substrates of AEPs [13].

In immune cells, the proteolytic process of TLR7/9 by AEPs is a critical step for the activation of an innate immune response and inflammatory reaction. In lymphoblastoid B cells, AEPs participate in the degradation of myelin basic protein (MBP) and thus its conversion into antigenic peptides that are associated with multiple sclerosis [34]. Recently, AEPs were found to cleave human α-synuclein in the brain and probably mediate pathological changes in Parkinson’s disease (PD) [17]. Moreover, AEPs process phosphatase 2A inhibitor SET, tau protein and amyloid precursor protein (APP) in neurons; therefore, they may play a vital role in the onset and progression of neurodegenerative diseases [17,35]. Most neurological disorders are incurable, and their pathogenesis is not clearly understood. In the following sections, this review briefly outlines the pathological changes mediated or driven by AEPs in Alzheimer’s and other neurological diseases on the basis of relevant reports and our recent studies.

## 4. AEPs in Alzheimer’s Disease (AD)

Dementias are cognitively impaired brain illnesses, 60–70% of which are AD, and the remaining cases consist of vascular dementia (VD), frontotemporal lobar degeneration (FTLD), dementia with Lewy bodies (DLB), and other neurodegenerative conditions [36]. AD is a progressive cognitive decline disorder with age-related prevalence divided into sporadic and familial AD types. In the world, over 90% of AD cases are of the sporadic form, which attacks after the age of 65, with incidence doubling every 5 years [37]. The familial AD accounts for 5% of AD cases and usually develops before the age of 65 (some patients begin in their 30s or 40s), mainly associated with mutations in APP gene on chromosome 21, presenilin-1 (PSEN1) on chromosome 14, and presenilin-2 (PSEN2) gene on chromosome 1 [36,38]. In the etiology of sporadic AD, many disease-causal lifestyle and genetic factors have been mentioned, such as age, pollution, smoking, obesity, diabetes, depression, oral diseases, brain injury, sleep disturbance, and the presence of the apolipoprotein E4 (APOE4) allele gene on chromosome 19, of which aging is the greatest risk factor [38,39]. AD, named in 1910, is currently the sixth leading cause of death in the United States due to a lack of effective methods to prevent, treat, or reverse cognitive impairment [36,40]. The etiopathogenesis of sporadic AD is very complex and has not yet been fully understood.

The damaging pathology of AD mainly includes polymorphous amyloid-β protein (Aβ) deposits in the brain and the formation of neurofibrillary tangles (NFTs) in neuronal cells, which are filamentous aggregates of hyperphosphorylated microtubule-associated protein tau. In an AD brain, tauopathy and neuritic plaque deposits, and the degeneration of cholinergic neurons gradually accumulate with age [39]. Why the development of these lesions is age-dependent has not been well-explained. Over the past few years, research on the role of AEPs in the pathogenesis of AD provides a breakthrough in answering this question. New evidence shows that the protein expression and activity of AEPs are progressively upregulated in murine brain along with aging. Previously, the pathogenic overexpression of AEPs had primarily been identified in the tumor microenvironment. Today’s studies and materials indicate that, in both aged and AD patient brains, there is a significantly increased level of AEP activation [41,42]. Upregulated AEPs probably drive the onset of sporadic AD through cleaving substrates SET, tau, and APP in aging brains, as demonstrated by rapidly growing data and evidence (see below).

**Figure 1:**
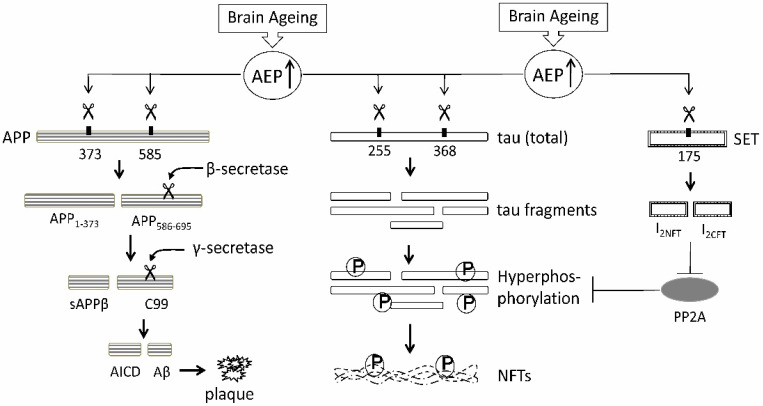
The pathogenic pathways of Alzheimer’s disease (AD) triggered by the asparaginyl endopeptidase (AEP). With brain aging, expression and activity of AEPs are upregulated. Overactivated AEPs cleave amyloid precursor proteins (APPs) at N373 and N585 residues. The cleavage of APPs at N585 removes steric hindrance between β-secretase enzymes and APPs, facilitating β-secretase to process APP586-695, subsequently leading γ-secretase to generating more Aβ. Concurrently, AEPs hydrolyze tau protein at N255 and N368 sites, generating neurotoxic fragments that elicit the hyperphosphorylation of tau and the formation of neurofibrillary tangles (NFTs). AEPs also digest SET, the inhibitor 2 (I_2_^PP2A^) of protein phosphatase 2A (PP2A), at Asn-175 into the N- and the C-terminal fragments (I_2NTF_ and I_2CTF_). Both I_2NTF_ and I_2CTF_ bind to the catalytic domain of PP2A; this binding represses the phosphatase activity of PP2A and promotes the hyperphosphorylation of tau. AEP↑: up-regulation of AEPs; 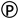: phosphorylation of tau.

First, the postmortem analysis of AD patients reported acidosis or reduced pH existing in brain tissue [43,44]. Such an acidic condition activates AEP and promotes its translocation to cytoplasm of neurons, where AEPs digest protein phosphatase 2A inhibitor 2, I_2_^PP2A^ (also named SET) at Asn-175 into the N-terminal fragment (I_2NTF_) and the C-terminal fragment (I_2CTF_) [42]. Both I_2NTF_ and I_2CTF_ bind to the catalytic domain of PP2A and inhibit its activity; this inhibition represses the phosphatase activity of PP2A and leads to the hyperphosphorylation of tau (Figure 1) [42]. Moreover, Zhang et al. reported that AEPs, having a skill similar to that of caspases, calpains, and thrombin, directly cleave tau proteins at N255 and N368 sites (Figure 1), generating neurotoxic tau fragments 1–368 and 256–368, which causes apoptosis in primary neurons; resultant fragments tau1–255 and 1–368 elicit the hyperphosphorylation of tau and the formation of NFTs [41]. Coincidentally, an in vitro mutation study revealed that the Asn-368 residue of tau was responsible for its aggregation induced by tau seeds that had been isolated from the brains of AD patients [45]. A report of cohort studies proved the presence of AEP-derived tau368 fragments in the brains of AD patients. This study employed a novel Simoa^®^ assay for the detection of tau368 in cerebrospinal fluid (CSF), and shows that the absolute levels of tau368 in CSF were significantly greater in AD patients than those in the control [46].

Experiments on animal models also demonstrated the pathophysiological role of AEPs in tauopathy, neuronal death, and behavioral deficits. In the tau P301S-transgenic mice of a tauopathy model, the ablation of the LGMN gene significantly suppressed the cleavage and hyperphosphorylation of tau, prevented a reduction in synapses and dendritic spines, and ameliorated cognitive impairment and memory loss [41]. Our recent study on senescence-accelerated mouse model SAMP8 revealed that the enzymatic activity of AEPs was elevated with age in the brain. In SAMP8 mice, the pharmacological inhibition of AEPs with a selective inhibitor suppressed tau processing and hyperphosphorylation, alleviated dendritic disruption and cognitive decline [15]. So, the age-dependent overactivation of AEPs is an enabling mechanism of brain tauopathy.

Second, the AEP translocates from endolysosomes into the cytoplasm and plasma membrane of neurons, where it cuts APPs at N373 and N585 residues through a pH-dependent manner, producing fragments that include APP1-373 and APP586-695 (Figure 1) [47]. The secretory APP1-373 is neurotoxic, inducing apoptosis and axonal damage in primary neurons. In human AD brain samples, proteomic analysis provisionally identified APP fragments terminating at N585 [47]. This AEP-mediated digestion of APPs at N585 may remove steric hindrance between β-secretase enzymes and APPs, facilitating β-secretase to process APP586-695 fragments, subsequently leading γ-secretase to produce more amount of Aβ [47]. In vitro, deletion of LGMN gene in primarily cultured neurons significantly reduced Aβ production. In vivo, knockout of LGMN gene from two animal models of AD, the 5XFAD and APP/PS1 mice, markedly decreased Aβ deposits and alleviated neuronal damage as well as cognitive decline [47].

Because of this newly identified secretase activity, AEPs were given one more name: δ-secretase enzyme [35,47]. Inspired by this new discovery, a relative specific AEP blocker compound 11 (δ-secretase inhibitor 11) was obtained that suppresses enzymatic activity of AEPs at nanomolar concentration range and can cross the blood–brain barrier (BBB) [48]. In AD models of tau P301S and 5XFAD mice, 3-month chronic treatment with δ-secretase inhibitor 11 significantly reduced tau cleavage and Aβ deposition, and ameliorated impairment in the structural and functional plasticity of synapses [48]. Moreover, treatment with δ-secretase inhibitor 11 suppressed inflammatory reaction, and reduced the secretion of IL-1β and TNFα in P301S and 5XFAD mice. Similarly, we treated SAMP8 and APP/PS1 mice with δ-secretase inhibitor 11 (10 mg kg^−1^, p.o.) for 3 months; the brain AEP activity of animal models was suppressed along with a significantly lesser deposit of Aβ and ameliorated memory loss [15,49]. Considering that the oral plasma elimination half-life (t_1/2_) of δ-secretase inhibitor 11 in mice is too short, about 2.31 h, although this AEP inhibitor is effective in treating animals, its practical significance in the application to human AD is very limited.

Third, the mechanisms through which AEPs are upregulated in aged brains are vital and have revealed a potential association with the CCAAT/enhancer binding proteins (C/EBPs), which belongs to a family of basic leucine-zipper (bZIP) transcription factors [50]. The C/EBPs family is composed of at least six members and their expression in cells is regulated by aging, cytokines, mitogens, nutrients and hormones [50,51]. Through the immunoblotting analysis of the brain of healthy humans and wild-type mice, Wang et al. found that the expression levels of both nuclear C/EBPβ and cytosolic AEPs in neurons progressively increased during aging. In addition, they revealed a more pronounced age-dependent increase in C/EBPβ and AEPs in the brain tissues of AD patients and mouse models [52]. In the mouse brain, C/EBPβ promoted LGMN mRNA transcription and increased AEP expression with age; the knock-out of the C/EBPβ gene completely abolished this age-dependent AEP upregulation. In transgenic AD model mice, C/EBPβ-triggered APP and tau cleavage, and AD-like pathology were all driven by upregulated AEPs. Accordingly, the inhibition of C/EBPβ reduced AEP expression, and decreased Aβ accumulation and tau hyperphosphorylation [52]. So, the activation of the neuronal C/EBPβ–AEP pathway during aging probably plays a dominant role in the onset of sporadic AD. Most recently, this mechanistic pathway was employed to explain why older women with dramatic hormonal changes are more likely to develop AD than men of the same age are [53]. Xiong et al. reported that the postmenopausal surge in serum follicle-stimulating hormone (FSH) contributed to a higher prevalence of AD in older women; elevated FSH bound to receptors on the surface of neurons, activating C/EBPβ–AEP cascades and leading to AD lesions including tauopathy and formation of Aβ plaques, synapse density reduction and cognitive impairment [53].

Collectively, the AEP, legumain, or δ-secretase is the only reported protease that concurrently regulates APP and tau metabolisms in the brain; the age-dependent activation of brain AEPs highly contributes to the pathogenesis of AD.

## 5. AEPs in AD Brain as a New Therapeutic Target and Relevant Concerns

The above-reported evidence indicates that the AEP-mediated processing of APP, tau, and SET constitutes upstream events of amyloid or tau toxicity; the pathological role of AEPs is vital for the initiation of early AD. Therefore, AEPs in the brain may represent an effective and promising drug target for the disease modification of AD. In the past, treatment strategies targeting amyloid or tau toxicity such as β-secretase inhibition, γ-secretase modulators, anti-Aβ antibodies and tau-targeted vaccines all failed in Phase 2 or 3 of clinical trials of AD [54]. These intervention methods were mostly designed towards the late disease stage of AD, which is an important cause of why previous efforts did not succeed [55]. If the AEP is established as a new therapeutic target, AEP inhibition strategies could be employed to intervene in the early stage of the disease and prevent disease progression, bringing a new chance of therapeutic success to AD. Nevertheless, before translating any AEP-targeting approaches into AD therapies, the following concerns should not be ignored.

First, conclusions on the role of AEPs in AD mostly come from the analysis of neurons or the whole brain tissue, but AEP expression is more abundant in microglia and astrocytes than it is in neurons [56,57]. Glial cells are an important source of inflammatory responses, and neuroinflammation is an underlying condition of AD [58,59,60]. In AD research, the role of AEPs in glial-cell-mediated neuroinflammatory reaction remains unexplored. Second, individuals with specific risk gene APOE4 have a higher chance of developing sporadic AD, but the mechanism for this increased risk remains to be elucidated [61,62]. Given the central role of AEPs in AD pathogenesis, is there any mechanistic relation between APOE4-incurred high AD incidence and AEP overactivation? Third, many AEP substrates exist in various organs; AEP inhibition strategies towards AD may inevitably cause side effects or even toxicity. For example, in a mouse model of unilateral ureteral obstruction, the pharmacological inhibition of AEPs with RR-11a exacerbated the progression of renal interstitial fibrosis following obstruction [63]. AEP inhibition may suppress the innate immune function, as the endogenous cleavage of TLR7 and TLR9 in dendritic cells (DCs) is dependent on AEPs [7,8,9,10,64]. Maschalidi et al. found that AEP-deficient mice infected by the influenza virus could not generate strong antiviral immune responses because AEP activity was critical for TLR7 processing and signaling [7]. In myeloid and plasmacytoid DCs stimulated with CpG, Sepulveda et al. found that AEPs were involved in TLR9 signaling and the inhibition of AEPs with MV026630 impaired secretion of interferon, TNF-α and IL-6 [8]. Recently, Anderson et al. observed the impacts of AEP inhibition on mitochondrial function and iron metabolism in macrophages [65].

Optimistically, the expression level and enzymatic activity of AEPs in healthy brains are far lower than those in peripheral organs. The pharmacological inhibition of upregulated AEPs in the brain could not markedly suppress the AEP function of other organs; thus, it may not generate side effects as apparent as the knockout of the AEP gene. AEP-null mice reproduce and grow up normally, but manifest hemophagocytic syndromes and age-related renal fibrosis [66,67,68]. On the other hand, a behavioral study found that AEP knockout mice displayed reduced anxiety, improved cognition abilities, and enhanced synaptic plasticity compared with wild-type mice [69], suggesting the psychiatric benefits of AEP inhibition.

## 6. AD Biomarkers and Functional Imaging of AEPs

AD is a progressive neurodegenerative disease with insidious onset. It often undergoes a 15–20 years of a preclinical or presymptomatic stage during which Aβ deposits, tau aggregates, and neuroinflammation reactions accumulate slowly and continuously with age, but not severely enough to cause symptoms [70]. If the risk factors of AD continue to ferment, asymptomatic individuals enter the stage of mild cognitive impairment (MCI) and have problems with memory, language, or judgment due to the gradual loss of neuronal synapses. The one-year conversion rate for MCI patients progressing into the dementia stage ranges from 10 to 18.4% according to different reports [71,72]. The preclinical or early stage of AD has become an important research focus in the area because it offer a chance for early diagnosis and timely intervention to arrest disease progression. Nevertheless, challenges remain in the definition of preclinical AD and standardization of biomarkers that can identify preclinical stage. Concentration of amyloid-β 42 (Aβ_42_) in CSF, phosphorylated tau (P-tau) in CSF, P-tau181 and P-tau231 in the plasma and Aβ positron-emission tomography (PET) imaging are helpful pathophysiological markers for detecting preclinical phase of the disease, but not yet recognized as specific biomarkers to confirm early diagnosis [73,74,75]. Other early signs of AD and dementia, such as sleep disorders, apathy, retinal amyloid imaging, and CSF tau368 and plasma P-tau217 levels are still being investigated [76,77,78,79]. Ideal biomarkers must be able to specifically indicate the presence of early pathological changes that are vital for AD onset.

The above discussion and argument indicate that upregulated AEPs and AEP-mediated APP and tau cleavage not only initiate AD, but are also upstream signals of amyloid or tau toxicity and thereby worthy of being explored as preclinical biomarkers. However, AEPs are widely distributed in various organs of the body, including the brain, which is isolated from the external environment by the BBB. The levels of AEPs and AEP-derived peptide fragments in body fluid are confused by different sources; thus, they are not proportional to their levels in the brain parenchyma. With the presence of the BBB, it is difficult to use body-fluid-based methods to measure brain AEPs. Fortunately, advances in bioimaging technologies suggest that this difficulty can be resolved through using AEP activity-based imaging probes and directly visualizing enzymatic activity in live subjects. Over the past 17 years, researchers have synthesized many types of AEP or legumain-targeted imaging probes, such as legumain inhibitors labeled with biotin and ^125^I radioisotope, inhibitors equipped with Cy5 fluorophores, and quenched activity-based legumain probes [80,81,82,83,84]. These probes had varying degrees of selectivity for AEPs, and were experimentally applied to visualize augmented AEP activity of tumor cells in live animals. Nevertheless, none of them has ever been used to perform imaging analysis of brain AEPs in the setting of neurodegeneration.

To perform the in vivo imaging analysis of brain AEP activity, we recently utilized gold nanoparticles (AuNPs) as carriers synthesized an aggregation reaction-based fluorescent AEP imaging probe, for which Cy5.5 conjugated AuNPs could be selectively activated by cellular AEPs and emit strong fluorescence [49]. This AEP-targeted probe had a general applicability in various cell lines, including glioma cells and degenerative neurons, and its florescence imaging intensity correlated well with the enzymatic activity of AEPs expressed in these cells. We applied this cell-permeable probe to a mouse model of AD and found that AEP imaging in a live brain was able to detect an upregulated AEP level at the early disease stage. For the first time, our work provided a proof of concept that the imaging assessment of brain AEP activity is a potential biomarker for the early diagnosis of AD. Further, the imaging analysis of brain AEP activity could be used to evaluate the efficacy of drugs that target AEPs as an AD treatment, since the pharmacological inhibition of AEPs is effective in preventing AD-like pathological progression in animal models.

Our AEP probes were based on a fluorescence imaging mechanism and tested on animals [49]. The avenue of developing new brain AEP imaging probes and methods is open. The BBB is the first barrier they need to cross. The invention of new AEP probes or contrast agents for computerized tomography (CT) and magnetic resonance imaging (MRI) is highly recommended because these approaches can be directly applied to AD patients to perform real-world studies. Experimentally, Yuan et al. designed a dual-functional AEP probe on the basis of the self-assembly and disassembly of ^19^F nanoparticles that confer ^19^F MRI signals “off” and “on”. They used this AEP-targeted MRI probe to image AEP activity in tumor-cell-bearing zebrafish [85]. One more example is a MRI contrast agent ([Gd-NBCB-TTDA-Leg(L)]) containing a AEP-specific substrate peptide that could be cut by AEPs, and produce increased hydrophobicity and rotational correlation time (τ(R)). This MRI contrast agent was used to image the AEP activity of tumor cells implanted in nude mice [86]. A recent study synthesized AEP-targeted ultrasound imaging probes to visualize implanted breast cancer cells in mice [87]. There are still no reports that have utilized CT or MRI imaging to analyze AEP activity in degenerative brain diseases.

## 7. AEPs in Other Neurological Diseases

**Figure 2:**
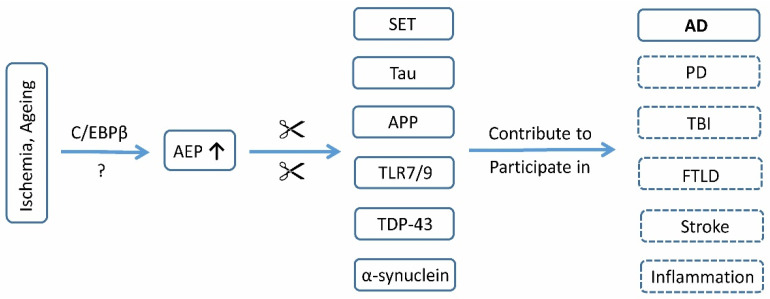
Involvement of AEPs in Alzheimer and other neurological diseases. Aging and ischemia upregulate AEPs in the brain, probably through activation of C/EBPβ or other transcriptional regulation mechanisms (?). The cleavable substrates of AEPs include SET, tau, APP, α-synuclein, TDP-43, and TLR7/9. Upregulated AEPs contribute to the pathogenesis of Alzheimer’s disease (AD), and may participate in the progression of Parkinson’s disease (PD), traumatic brain injury (TBI), frontotemporal lobar degeneration (FTLD), stroke, and neuroinflammation.

AEP upregulation and the consequent deleterious reactions are not unique to AD. In the secondary phase of acute brain injuries, such as traumatic brain injury (TBI) and stroke, AEPs are upregulated and involved in postneurotrauma neurodegeneration (Figure 2). In TBI models induced by the controlled cortical impact, Wu et al. found that AEPs were elevated and maintained at higher levels from 6 h to 6 months after the induction of TBI [88]. Followed by AEP upregulation, AEP-mediated tau and APP cleavage was escalated at Day 7, and tau hyperphosphorylation and Aβ deposition were observed at 6 months after TBI induction. Additionally, TBI-incurred neuroinflammatory responses such as the activation of microglia, and the overexpression of IL-1β, IL-6 and TNFα were closely associated with AEP upregulation [88]. In stroke models induced by the transient occlusion of the middle cerebral artery (tMCAO), a time-dependent increase in AEP immunoreactivity was identified in the peri-infarct region from 2 to 7 days after tMCAO [89]. AEP upregulation was involved in the activation and invasion of inflammatory CD74^+^ microglia in the brain area affected by ischemia [89]. Brain ischemia also leads to tau hyperphosphorylation through the activation of AEPs, the cleavage of SET, and the inhibition of PP2A [90]. In vitro, oxygen–glucose deprivation (OGD) exposure augmented AEP activity in cultured neuronal cells [91]. In TBI and ischemic brains, AEP upregulation probably results from the activation of transcription factor C/EBPβ or nuclear calcium (Ca^2+^) signals [88]. TBI or stroke increases the risk of developing AD and dementia; AEPs could contribute to this disease conversion.

TAR DNA-binding protein 43 (TDP-43), α-synuclein, and UNC5C receptors are late-identified substrates of AEPs (Figure 2). Nuclear protein TDP-43 is the major disease-associated protein implicated in the pathogenesis of FTLD [92]. In the brain, AEPs cut TDP-43 at N291, N306, and five other sites [93]. TDP-43 mutations associated with FTLD may affect AEP-mediated cleavage. Etiopathogenetic studies of PD revealed that AEPs cleaved human α-synuclein and UNC5C receptors in an age-dependent manner, leading to the degeneration of dopaminergic neurons and motor function deficits [94,95]. Recently, Lin et al. found that AEPs were overexpressed in glioblastoma (GBM), and the augmented tumorigenesis of GBM via inactivating P53-mediated tumor suppression [96]. Developing the AEP into a biomarker of AD requires differential diagnosis with TBI, stroke, FTLD, PD and GBM, which can be resolved through symptoms, disease history, physical examining, CT and MRI scanning.

## 8. Conclusions

Below are key points regarding involvements of the AEP in neurodegenerative diseases.

First, the expression and enzymatic activity of AEPs in healthy brains are far lower than those of peripheral organs; aging and other stresses may upregulate and overactivate brain AEPs. The abnormal upregulation of AEPs in a certain brain region could be an important indicator of neurological damage. Aging does not always upregulate AEPs, just as aging does not always lead to AD, relating to a matter of probability. AEP-mediated SET, APP, and tau cleavage are mechanisms through which aging or other factors increase the incidence of AD.

Second, therapeutic strategies targeting AEPs are suitable for chronic degenerative diseases such as AD, but not good for the acute treatment of TBI or stroke because the AEP does not seem to participate in acute stage injury. However, following the acute phase of TBI or stroke, secondary chronic neurodegeneration often occurs, which involves AEP upregulation; thus, it may be intervened with AEP inhibition strategy.

Third, the above experimental evidence suggests that AEPs are an emerging drug target for the disease modification of AD; the imaging assessment of AEPs in the live brain is a potential biomarker for predicting the preclinical stage. However, more evidence from AD patients is required, because clinical data supporting this conclusion are currently not sufficient. In addition, the inhibition of AEPs may have potential immunosuppressive effects and affect renal function. To promote progress in this area, it is time to develop new AEP inhibitors or candidate drugs with acceptable druggability to perform translational research, and invent advanced AEP-targeted imaging probes to evaluate the predictive efficacy of brain AEP imaging as a biomarker for preclinical AD, particularly in a real-world setting.

## Data Availability

The data supporting the findings of this study are available within the article and from the corresponding author upon request.

## Abbreviations

AD	Alzheimer’s disease
AEP	asparaginyl endopeptidase
LGMN	legumain
APP	amyloid precursor proteins
Aβ	amyloid-β protein
Asn	asparagine
Asp	asparate
TLRs	toll-like receptors
APCs	Ag-presenting cells
ER	endoplasmic reticulum
LSAM	Legumain stabilization and activity modulation
MBP	myelin basic protein
PD	Parkinson’s disease
VD	vascular dementia (VD)
FTLD	frontotemporal lobar degeneration (FTLD)
DLB	dementia with Lewy bodies
APOE4	apolipoprotein E4
NFTs	neurofibrillary tangles
SET	an inhibitor of protein phosphatase 2A
PP2A	protein phosphatase 2A
C/EBPβ	CCAAT/enhancer binding protein β
FSH	follicle-stimulating hormone
MCI	mild cognitive impairment
CSF	cerebrospinal fluid
P-tau	phosphorylated tau
PET	positron-emission tomography
BBB	blood–brain barrier
AuNPs	gold nanoparticles
CT	computerized tomography
MRI	magnetic resonance imaging
TBI	traumatic brain injury
tMCAO	transient occlusion of the middle cerebral artery
OGD	the oxygen–glucose deprivation
Ca^2+^	calcium
TDP-43	TAR DNA-binding protein 43
GBM	glioblastoma
